# miR-377 inhibition enhances the survival of trophoblast cells via upregulation of FNDC5 in gestational diabetes mellitus

**DOI:** 10.1515/med-2021-0247

**Published:** 2021-03-25

**Authors:** Zhaozhao Hua, Dana Li, Anqin Wu, Ting Cao, Shi Luo

**Affiliations:** Department of Obstetrics, The Second Affiliated Hospital of Guizhou University of Traditional Chinese Medicine, No. 83 Feishan Street, Yunyan District, Guiyang City, Guizhou Province, 550003, China

**Keywords:** gestational diabetes mellitus, miR-377-3p, FNDC5, cell survival, trophoblast cells

## Abstract

Gestational diabetes mellitus (GDM) is a metabolic dysregulation closely related to both obesity and type 2 diabetes; however, the molecular mechanism underlying GDM is still unclear. The purpose of this study was to investigate the effects of microRNA-377 (miR-377-3p) and fibronectin type III domain containing 5 (FNDC5) in regulating the cell growth of trophoblasts under high glucose (HG) conditions during the development of GDM. Serum miR-377-3p was upregulated and positively correlated with fasting blood glucose level in GDM patients. miR-377-3p downregulation increased the cell vitality and suppressed the cell apoptosis of HG-treated HTR-8/SVneo and BeWo cells. Using TargetScan prediction, luciferase assay, and western blot, it was found that miR-377-3p could target FNDC5 and suppress its expression. However, FNDC5 downregulation abolished the effect of miR-377-3p inhibitor in HTR-8/SVneo cells. Together, miR-377 is a potential target for GDM biomarker, which promotes cell growth and suppresses cell apoptosis, partly through the upregulation of FNDC5.

## 1 Introduction

Gestational diabetes mellitus (GDM) is a metabolic dysregulation closely related to both obesity and type 2 diabetes [1]. Pregnant women may suffer from impaired islet function, abnormal blood glucose metabolism, pregnancy hypertension, leading to adverse pregnancy outcomes such as respiratory distress syndrome and wet lung [2]. Cardiovascular abnormalities and metabolic syndrome may occur in the offspring of gestational diabetes patients, and the incidence and mortality of respiratory distress syndrome are elevated. The intellectual and behavioral developments in neonatal and childhood stages are also affected, and the risk of obesity and diabetes in long-term adolescence is also increased [3]. In recent years, the research on GDM has attracted great attention, but the molecular mechanism underlying GDM is still unclear.

**Table 1:** The expression of FoxO1 in placenta and omental adipose tissue of GDM

Characteristics	Health	GDM	*p-*value
Number	38	30	
Age (years)	33.21 ± 8.17	30.27 ± 6.38	0.1098
Gestational weeks	37.54 ± 1.31	38.12 ± 1.65	0.1119
Height (m)	1.61 ± 0.06	1.59 ± 0.56	0.2123
Pre-pregnancy weight (kg)	50.75 ± 5.85	50.1 ± 6.02	0.6526
Current weight (kg)	68.24 ± 6.40	70.35 ± 6.52	0.1848
Weight gain during pregnancy (kg)	17.48 ± 8.16	20.25 ± 7.12	0.1467
Pre-pregnancy BMI (kg/m^2^)	19.57 ± 2.63	19.78 ± 2.91	0.7713
Current BMI (kg/m^2^)	25.68 ± 2.52	28.00 ± 3.47	0.0503
Birth weight (g)	3518.98 ± 558.15	3545.05 ± 587.15	0.8523
Fasting plasma glucose (FPG)	3.72 ± 0.88	4.96 ± 1.59	0.0001*
Fasting insulin (FIN)	8.76 ± 2.10	15.70 ± 4.62	0.0001*
Homeostasis model of assessment-insulin resistance (HOMA-IR)	1.45 ± 0.30	5.50 ± 1.66	0.0001*
miR-377 expression	1.53 ± 1.11	2.49 ± 1.70	0.0068*

*p < 0.05.

Using targeting gene’s 3′-untranslated region (UTR), microRNAs (miRNAs) usually post-transcriptionally regulate gene expression. More and more miRNAs have been found to play important roles in the pathogenesis of diabetes. In particular, the role of some miRNAs in gestational diabetes has recently been found [4]. Studies have shown that high glucose (HG) can induce human umbilical vein endothelial cell dysfunction via upregulation of miR-137 levels in gestational diabetes [5]. miR-657 promoted macrophage polarization to M1 by targeting FAM46C (family with sequence similarity 46, member C) in gestational diabetes [6]. miR-137 restricts the survival and migration of human chorionic trophoblast cells HTR-8/SVneo cells by decreasing fibronectin type III domain containing 5 (FNDC5) in gestational diabetes [7]. miR-503 is upregulated in placental tissues and peripheral blood of GDM patients, and miR-503 can target FNDC5 to disrupt the function of islet cells [8]. Studies have shown that miR-377-3p overexpression can upregulate the level of fibronectin in diabetic nephropathy [9]. However, the role and mechanism of miR-377-3p in gestational diabetes have not been reported.

In this study, serum miR-377-3p level and its correlation with fasting blood glucose of the GDM patients were examined and analyzed. miR-377-3p’s effects on trophoblastic cell survival and apoptosis under the condition of HG were investigated. The targeting relationship between miR-377-3p and FNDC5 was studied using TargetScan prediction and western blotting. In addition, the effects of FNDC5 downregulation on cell growth and apoptosis of HTR-8/SVneo cells mediated by miR-377-3p inhibitor were analyzed.

## 2 Methods

### 2.1 Clinical samples

All serum samples were collected from the Second Affiliated Hospital of Guizhou University of Traditional Chinese Medicine. A total of 30 GDM pregnant patients and 38 normal pregnant women (healthy) after C-section in the third trimester of gestation were enrolled (Table 1). GDM was diagnosed according to the criteria published by the International Association of the Diabetes and Pregnancy Study Groups (IADPSG) [10]. Some cases were excluded, which included women with *in vitro* fertilization (IVF), preeclampsia, twins (multiple) pregnancy, maternal diabetes history, or other pregnancy complications. All serum samples were frozen by liquid nitrogen and stored at −80°C.


**Ethics approval:** This study was approved by the Ethics Committee of the Second Affiliated Hospital of Guizhou University of Traditional Chinese Medicine.
**Statement of informed consent:** Written informed consent was obtained from a legally authorized representative(s) for anonymized patient information to be published in this article.

### 2.2 RNA extraction and quantitative reverse transcription PCR (qRT-PCR)

Total RNAs in the serum and cells were extracted by TRIzol (catalog number, 15596018, Thermo Fisher Scientific, Waltham, MA, USA). For reverse transcription, the Prime Script RT Reagent Kit (catalog number, #RR037A, Takara) was used. The cDNAs were obtained and then amplified with the ExTaq Kit (catalog number, DRR001A, Takara) to determine the miRNA levels. The U6 small nuclear RNA (snRNA) was used to normalize miR-377-3p expression. The primer sequences were: miR-377-3p (forward): 5′-GAGCAGAGGTTGCCCTTG-3′, miR-377-3p (reverse): 5′-ACAAAAGTTGCCTTTGTGTGA-3′; U6 (forward): 5′-CTCGCTTCGGCAGCACA-3′, U6 (reverse): 5′-AACGCTTCACGAATTTGCGT-3′.

### 2.3 Cell culture

Human trophoblast cell line HTR-8/SVneo and BeWo were purchased from the Chinese Academy of Sciences Cell Bank (Shanghai, China). HTR-8/SVneo cells were derived by transfecting the cells that grew out of chorinic villi explants of human first-trimester placenta with the gene encoding for simian virus 40 large T antigen. As a derivative of malignant choriocarcinoma, BeWo cells preserved functional hormone synthesis in and cell morphology of the cytotrophoblast of the original tumor. These two cell lines share a number of phenotypic properties with the parental trophoblast cells and are useful to study trophoblast and placental biology. Dulbecco’s modified Eagle’s medium (catalog number, 30030, DMEM, Thermo Fisher Scientific) supplemented with 10% fetal bovine serum (catalog number, 16140071, FBS, Thermo Fisher Scientific) was used for cell culture, which was maintained in a humidified atmosphere with 5% CO_2_ at 37°C. The cells in the control group were cultured in the basal medium with a glucose concentration of 5 mM, whereas cells in the HG group were incubated in a HG medium (glucose concentration of 25 mM).

### 2.4 Cell transfection

Cell transfection reagents including miR-377-3p inhibitor, miR-377-3p mimic, and the separate negative controls (NC inhibitor or NC mimic) were synthesized (GenePharma, Shanghai, China) to study the effect of miR-377-3p. These mimic and inhibitor were respectively transfected into the trophoblast cells with the help of Lipofectamine 2000 (catalog number, 11668019, Thermo Fisher Scientific) according to the manufacturer’s instructions. After transfection, the cells were used for the qRT-PCR, cell viability, apoptosis assay, and western blot assays.

### 2.5 Cell viability assay

The HTR-8/SVneo and BeWo cells were transfected with miR-377-3p inhibitor or NC inhibitor for 24 h. Briefly, cells (5,000 per well) were seeded into 96-well plates with basal or HG culture medium. After that, cell viability was determined using cell counting kit-8 (catalog number, CK04, CCK8, Dojindo, Japan). The absorbance at 450 nm was detected using a microplate reader (Molecular Devises, CA, USA).

### 2.6 Apoptosis assay

At 48 h post transfection, apoptosis assay was performed in HTR-8/SVneo and BeWo cells. The cells were first fixed with 70% ethanol overnight and were treated with propidium iodide (PI), Annexin V (catalog number, 556570, BD Biosciences, San Jose, CA). After that, apoptosis rate was analyzed using a FACS Calibur system (BD Biosciences).

### 2.7 Luciferase reporter assay

To determine the direct interactions between miR-377-3p and FNDC, luciferase reporter assay was performed. First, DNA fragments of the FNDC 3′-UTR that contain miR-377-3p binding sites were amplified. Then, the 3′-UTR of FNDC was cloned into the pMIR-Report luciferase vector (catalog number, VT1399, Promega, Madison, WI, USA). A vector with mutated miR-377-3p putative binding site was used as a control. After co-transfection with pMIR-Report vectors, β-gal (a reference for transfection efficiency) and miR-377-3p mimic (NC mimic), luciferase activity was determined by a dual luciferase reporter assay system (catalog number, E1960, Promega).

### 2.8 Protein extraction and western blot

Western blotting was performed as previously reported [11]. Total protein from HTR-8/SVneo and BeWo cells was extracted by RIPA buffer (catalog number, 310003, BestBio, Shanghai, China). Protein samples were separated using sodium dodecyl sulfate-polyacrylamide gel electrophoresis (SDS-PAGE) and transferred onto polyvinylidene difluoride (PVDF) membranes (Bio-Rad Laboratories, Richmond, CA, USA). After blocking with 5% non-fat milk, the membranes were incubated with the primary antibody of FNDC5 (catalog number, 23995-1-AP, Thermo Fisher Scientific, dilution ratio 1:2,000) overnight at 4°C. Secondary goat anti-rabbit IgG antibodies conjugated with horseradish peroxidase (catalog number, A27036, Thermo Fisher Scientific, dilution ratio 1:2,000) were used. β-actin (catalog number, MA1-140, Thermo Fisher Scientific, dilution ratio 1:2,000) expression was used as control. For protein visualization, an ECL Kit (catalog number, 345818, Millipore, Billerica, MA, USA) and a chemiluminescence imaging system were used. The western blot bands were analyzed by Image J software (https://imagej.net/Citing).

### 2.9 Statistical analysis

Data are expressed as mean ± standard error of the mean (SEM). Statistical differences between two groups were analyzed using Student’s *t*-test. One-way ANOVA was used for the analysis of statistical differences among the experimental groups. *p* < 0.05 was considered statistically significant.

## 3 Results

### 3.1 Serum miR-377-3p is significantly increased in GDM patients

**Figure 1:**
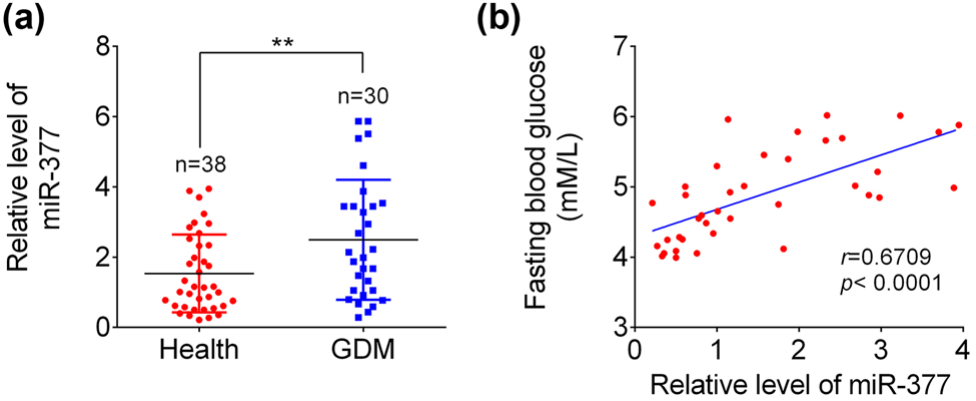
Expression of miR-377-3p and its correlation with fasting blood glucose in GDM patients. (a) Serum expression of miR-377-3p was upregulated in GDM patients compared with that in the healthy women. (b) Serum miR-377-3p levels were positively correlated with the fasting blood glucose in GDM patients (*r* = 0.6709, *p* < 0.0001). The data are expressed as mean ± SEM; ** indicates *p* < 0.01.

As shown in Figure 1a, qRT-PCR results demonstrated that serum miR-377-3p levels were much higher in GDM patients (*n* = 30) than those in the healthy pregnant women (*n* = 38). In addition, a positive correlation between relative level of miR-377-3p and fasting blood glucose level in GDM patients was found (Figure 1b), which suggested that miR-377-3p might play an important role in the pathogenesis of GDM.

### 3.2 miR-377-3p downregulation increases cell vitality and suppresses apoptosis of HG-treated HTR-8/SVneo and BeWo cells

**Figure 2:**
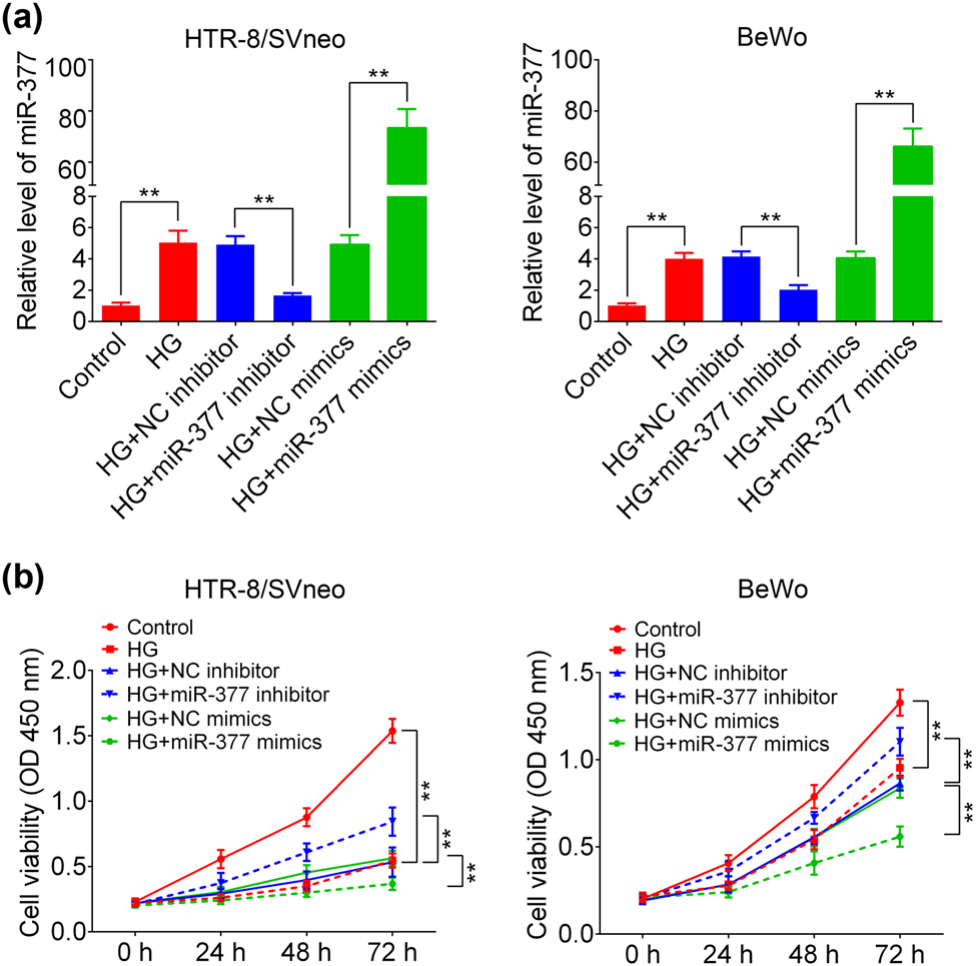
Effect of miR-377-3p on HTR-8/SVneo and BeWo cells. (a) miR-377-3p expression in HTR-8/SVneo and BeWo cells (control or HG) with transfection of miR-377-3p inhibitor, miR-377-3p mimic, NC mimic or NC inhibitor. (b) Cell growth curves of HTR-8/SVneo and BeWo cells (control or HG) with transfection of miR-377-3p inhibitor, miR-377-3p mimic, NC mimic or NC inhibitor. The data are expressed as mean ± SEM. ** indicates *p* < 0.01.

**Figure 3:**
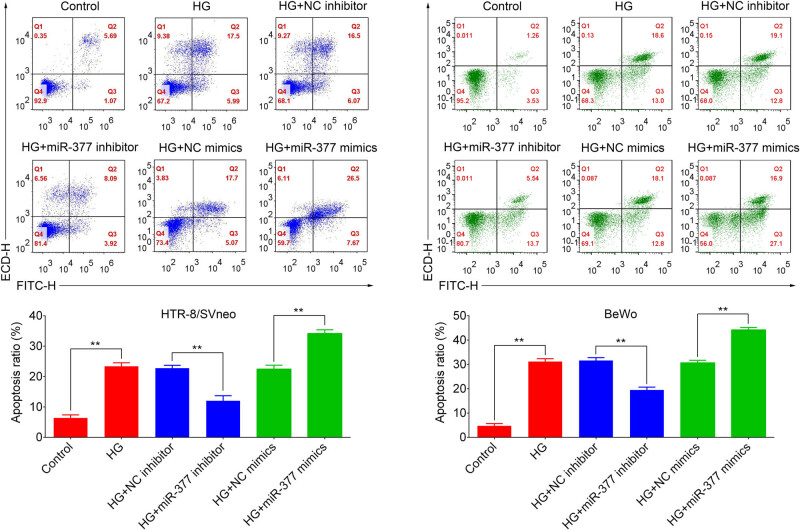
Apoptosis analysis of HTR-8/SVneo and BeWo cells (control or HG) and HG cells with the transfection of miR-377-3p inhibitor, miR-377-3p mimic, NC mimic or NC inhibitor. Apoptosis rates were quantified and shown as histograms. The data are expressed as mean ± SEM; ** indicates *p* < 0.01.

To verify the role of miR-377-3p in regulating the biological function of trophoblast cells under the condition of hyperglycemia, HTR-8/SVneo and BeWo cells were treated with HG medium for 24 h *in vitro*. As shown in Figure 2a, miR-377-3p level was significantly upregulated in the HG group, in contrast with that in control groups, suggesting a positive relation between miR-377-3p expression and glucose. miR-377-3p inhibitor resulted in a significant downregulation of miR-377-3p in HTR-8/SVneo and BeWo cells with or without HG treatment, and miR-377-3p mimic increased miR-377-3p level. In addition, the cell viability curves in Figure 2b demonstrate that HG treatment inhibited cell growth in HTR-8/SVneo and BeWo cells, whereas miR-377-3p inhibitor increased cell growth in HG-treated HTR-8/SVneo and BeWo cells. Furthermore, the apoptosis analysis in Figure 3 show that HG treatment upregulated the apoptosis ratio of HTR-8/SVneo and BeWo cells. Meanwhile miR-377-3p inhibitor reduced the apoptosis ratio in HG-treated HTR-8/SVneo and BeWo cells, whereas miR-377-3p mimic led to the opposite result of cell growth and apoptosis.

### 3.3 miR-377-3p targets FNDC5 in HTR-8/SVneo and BeWo cells

**Figure 4:**
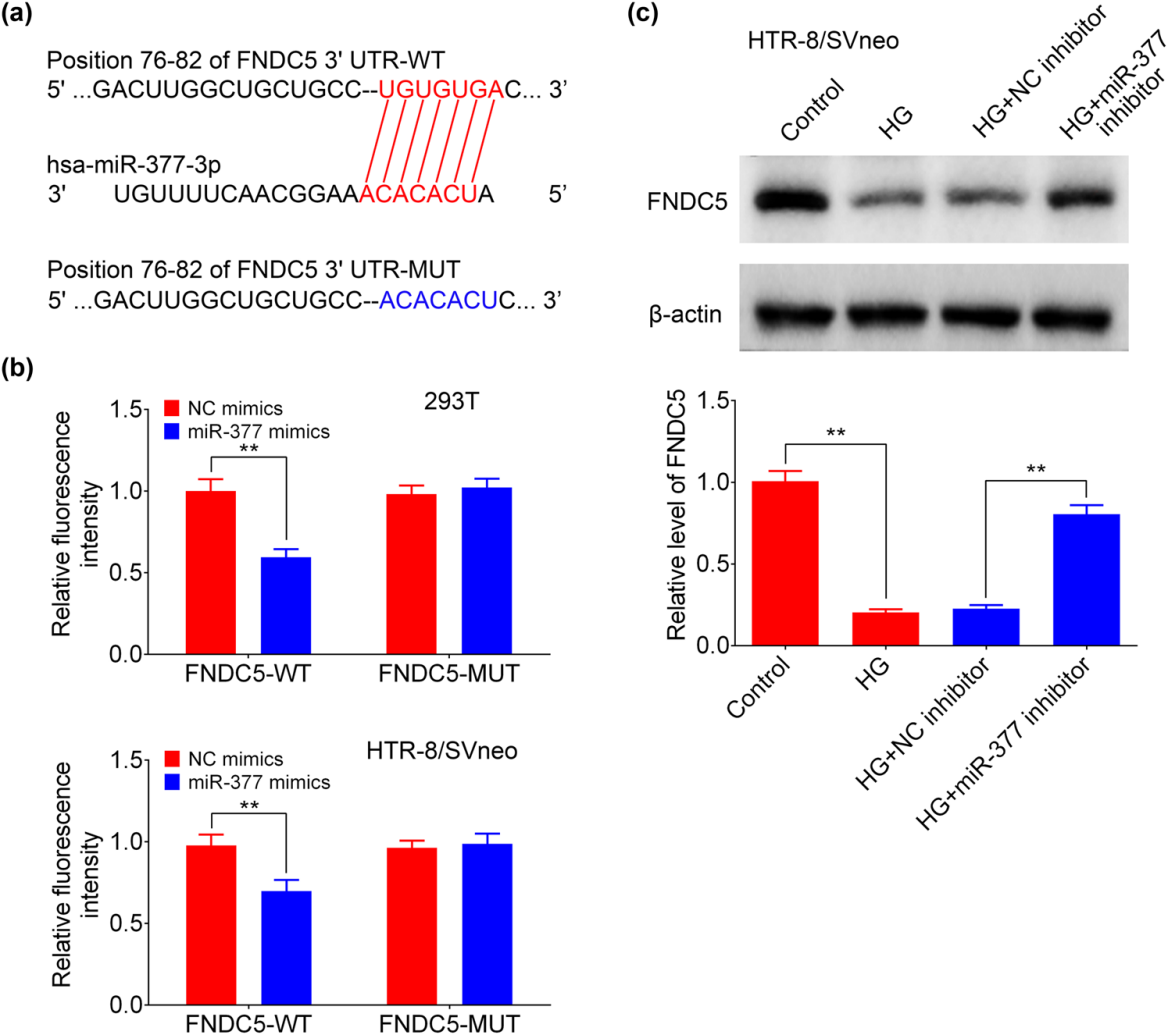
miR-377-3p negatively regulated FNDC5 expression. (a) The predicted seed-recognition sites in the 3′-UTR of FNDC5 mRNA and the miR-377-3p sequences were shown. (b) Relative luciferase activity of the FNDC5 3′-UTR reporter plasmid in 293 T and HTR-8/SVneo cells after transfection with NC mimic or miR-377-3p mimic. The mutant FNDC5 3′-UTR reporter was also used as a control. (c) Protein expression levels of FNDC5 in HTR-8/SVneo and BeWo cells from groups of control, HG, HG + NC inhibitor, HG + miR-377-3p inhibitor, HG + NC mimic, HG + miR-377-3p mimic, as determined using western blotting. Bands were quantified and shown in histogram. The data are expressed as mean ± SEM. ** indicates *p* < 0.01.

The results above indicated that miR-377-3p inhibition allows cell growth and decreases apoptosis level in trophoblastic cells under the condition of hyperglycemia. Thus, the molecular mechanism was investigated next. As predicted by TargetScan, an online prediction software, FNDC5, was found to be a potential target gene of miR-377-3p. Potential binding sites between miR-377-3p and FNDC5 are shown in Figure 4a. The data of the luciferase reporter experiment demonstrated that miR-377-3p mimic reduced the luciferase activity of FNDC5 3′-UTR reporter plasmid (Figure 4b). The protein expression of FNDC5 in HG-treated HTR-8/SVneo and BeWo cells was significantly downregulated. In addition, FNDC5 expression was markedly increased in the HG + miR-377-3p inhibitor group when compared to that in the HG + NC inhibitor group, whereas miR-377-3p mimic further downregulated the expression of FNDC5 under HG treatment (Figure 4c). The data above indicated that miR-377-3p targeted FNDC5 and suppressed its expression.

### 3.4 FNDC5 downregulation eliminates the effect of miR-377-3p inhibitor

**Figure 5:**
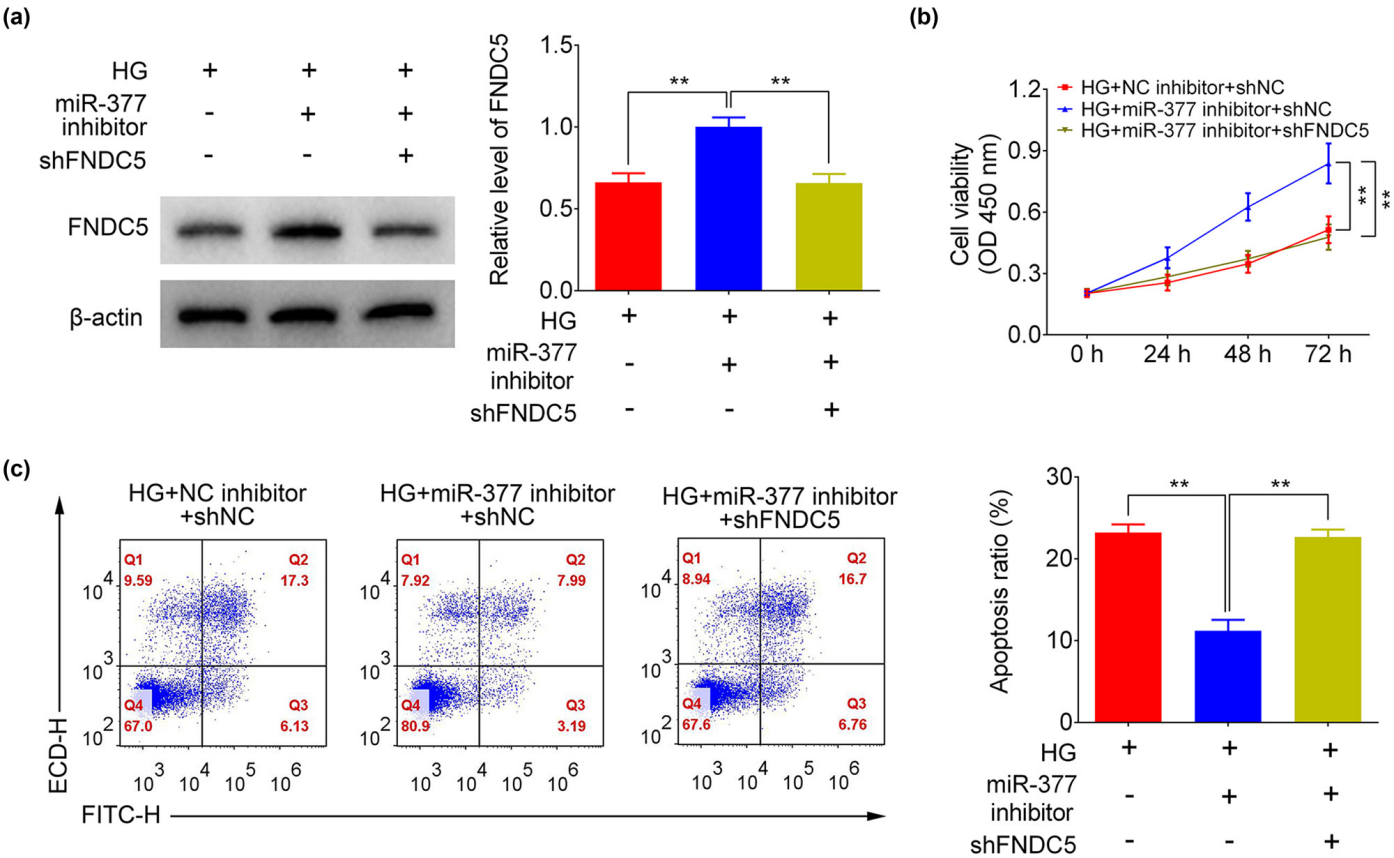
FNDC5 downregulation abolished the effects of miR-377-3p on cell proliferation and apoptosis. (a) Protein expression levels of FNDC5 in HTR-8/SVneo cells from groups of HG, HG + miR-377-3p inhibitor, HG + shFNDC5, or HG + miR-377-3p inhibitor + shFNDC5, as determined using western blotting. Bands were quantified and shown in histogram. (b) Cell growth curves of HTR-8/SVneo cells from groups of HG, HG + miR-377-3p inhibitor, HG + shFNDC5, HG + miR-377-3p inhibitor + shFNDC5. (c) Apoptosis analysis of HTR-8/SVneo cells (control or HG) and HG cells from groups of HG, HG + miR-377-3p inhibitor, HG + shFNDC5, HG + miR-377-3p inhibitor + shFNDC5. Apoptosis rates were quantified and shown as histograms. The data are expressed as mean ± SEM; ** indicates *p* < 0.01.

After establishing FNDC5 as a target gene of miR-377-3p, the role of FNDC5 in growth and apoptosis of HTR-8/SVneo cells was explored. As shown in Figure 5a, miR-377-3p inhibition caused an upregulation of FNDC5 in HG-treated HTR-8/SVneo cells, and the FNDC5 expression was repressed again by transfection of shFNDC5. The FNDC5 expression was further inhibited when transfecting shFNDC5 and miR-377-3p mimic. In addition, the increased cell viability caused by miR-377-3p inhibition in HG-treated HTR-8/SVneo cells were suppressed in cells of the HG + miR-377-3p inhibitor + shFNDC5 group (Figure 5b). Furthermore, miR-377-3p inhibition significantly downregulated the cell apoptosis ratio after HG stimulation. The reduced-cell apoptosis ratio was reversed in HG + miR-377-3p inhibitor + shFNDC5 group compared to that in HG + miR-377-3p inhibitor + shNC group (Figure 5c). Considering these findings, it can be concluded that upregulation of FNDC5 was required in cell growth promotion and apoptosis suppression mediated by miR-377-3p inhibitor in trophoblast cells, indicating its involvement in the development of GDM.

## 4 Discussion

As a health problem among pregnant women, GDM exerts negative consequences in pregnancy. The normal biological function of trophoblast cells is critical to the development of placenta. The reduced viability of trophoblast cells might cause dysplasia of pregnancy and even miscarriage [12]. miRNAs are sensitive and stable, and have been demonstrated to be potential diagnostic markers and intervention targets for human diseases, including diabetes mellitus [13] and also GDM [14]. For example, serum miR-204 was found to be upregulated in type 1 diabetes (T1D) patients and negatively correlated with β cell function [15]. So far, decreased miR-132 was found in GDM patients, and overexpression of miR-132 in HTR-8/SVneo cells could markedly rescue HG-induced suppression of cell proliferation [16]. More vital miRNAs and their roles in the pathogenesis of GDM deserve further investigation. This study showed that serum miR-377-3p was increased in the GDM patients, and a positive correlation between miR-377-3p level and the fasting blood glucose was indicated in GDM patients.

HG, as a characteristic of GDM, could inhibit proliferation of trophoblast cells, thereby impairing the development of placenta [17]. Consistently, cell viability of HTR-8/SVneo cells was significantly suppressed by the HG treatment in the present study, along with the upregulation of miR-377-3p. Interestingly, considerable effects were observed in early and late apoptosis in BeWo treated with HG + miR-377 mimics in contrast to HTR-8/SVneo cells where only late apoptosis was increased (Figure 3). The difference in early and late apoptosis ratios between early and late apoptosis percentages among HTR-8/SVneo and BeWo cells may account for their different sensitivity to HG treatment and the rate of renewal iteration. While the importance of miR-377-3p has been demonstrated in some tumors, it was found that miR-377-3p plays an oncogenic function in colorectal cancer development through increasing GSK-3β expression and thereby activating NF-κB pathway [18]. In addition, tumor inhibitory functions of miR-377-3p were demonstrated in pancreatic cancer by regulating a serine/threonine kinase, namely Pim-3 proto-oncogene [19], and in gastric cancer through reducing the level of vascular endothelial growth factor A (VEGFA) [20]. However, the role of miR-377-3p in diabetes is still poorly understood. Although serum miR-377-3p was reported to be higher in pediatric patients with T1D [21], upregulation of miR-377-3p in diabetic nephropathy indirectly resulted in the upregulation of fibronectin protein [9]. However, the function of miR-377-3p in GDM development still remains unclear. Herein, the first evidence for a protective function of targeting miR-377-3p was presented: miR-377-3p inhibition can restore the protein expression of FNDC5, reestablish cell growth, and reduce apoptosis ratio in HTR-8/SVneo and BeWo cells.

As a transmembrane protein present in various tissues including heart, liver, skeletal muscle, and adipose tissue, FNDC5, the precursor of irisin, was found to be a novel player in metabolism and metabolic syndrome [22]. Clinical studies combined with cellular experiments revealed that FNDC5 mRNA was decreased in adipose tissue of patients with type 2 diabetes [23]. In animal models of obesity, upregulation of FNDC5 increased uncoupling protein 1 (UCP1) expression and oxygen consumption, leading to high energy expenditure [22]. However, the potential mechanism that regulates FNDC5 in GDM needs to be further explored. In this study, it was found that miR-377-3p could directly target FNDC5 and its inhibition reestablish cell growth in HG-treated HTR-8/SVneo and BeWo cells. In addition to miR-377-3p, miR-137 was reported to suppress the viability and migration of trophoblasts through negatively regulating FNDC5 in GDM, which may result in adverse pregnancy outcomes [7]. Furthermore, myostatin (Mstn) could increase miR-34a, leading to downregulation of FNDC5, which inhibits the browning of white adipocytes [24]. Therefore, whether miR-377-3p, miR-137, and miR-34a could coordinately regulate FNDC5 expression in GDM, and how to synthetically adjust their levels to prevent and treat GDM require future research.

In brief, serum miR-377-3p is upregulated in GDM patients and positively correlates with the fasting blood glucose, which might serve as a potential diagnostic biomarker for GDM patients. The downregulation of miR-377-3p can relieve effects of HG on trophoblast cell proliferation and apoptosis, which is mediated through the regulation of FNDC5 expression. In addition, inhibition of FNDC5 could abrogate the effects caused by miR-377-3p inhibitor, indicating that miR-377-3p inhibition may have a beneficial role in GDM remission by reestablishing cell growth and reducing apoptosis ratio. Together, miR-377 may be a potential target for GDM biomarker.
